# X Chromosome Activity in Mouse XX Primordial Germ Cells

**DOI:** 10.1371/journal.pgen.0040030

**Published:** 2008-02-08

**Authors:** Susana M Chuva de Sousa Lopes, Katsuhiko Hayashi, Tanya C Shovlin, Will Mifsud, M. Azim Surani, Anne McLaren††

**Affiliations:** The Wellcome Trust/Cancer Research UK Gurdon Institute, University of Cambridge, Cambridge, United Kingdom; University of Cambridge, United Kingdom

## Abstract

In the early epiblast of female mice, one of the two X chromosomes is randomly inactivated by a *Xist*-dependent mechanism, involving the recruitment of Ezh2-Eed and the subsequent trimethylation of histone 3 on lysine 27 (H3K27me3). We demonstrate that this random inactivation process applies also to the primordial germ cell (PGC) precursors, located in the proximal region of the epiblast. PGC specification occurs at about embryonic day (E)7.5, in the extraembryonic mesoderm, after which the germ cells enter the endoderm of the invaginating hindgut. As they migrate towards the site of the future gonads, the XX PGCs gradually lose the H3K27me3 accumulation on the silent X chromosome. However, using a GFP transgene inserted into the X chromosome, we observed that the XX gonadal environment (independently of the gender) is important for the substantial reactivation of the inactive X chromosome between E11.5 and E13.5, but is not required for X-chromosome reactivation during the derivation of pluripotent embryonic germ cells. We describe in detail one of the key events during female PGC development, the epigenetic reprogramming of the X chromosome, and demonstrate the role of the XX somatic genital ridge in this process.

## Author Summary

The last few years have led to striking advances in our understanding of the genesis of primordial germ cells (PGCs) and the importance of their correct epigenetic programming for the formation of functional gametes in mice. We investigated one aspect of the epigenetic programming of germ cells, the activity of the XX chromosomes in female germ cells between the formation of PGC precursors and sex determination. Random inactivation of one of the X chromosomes occurs in all cells of the embryo including the PGC precursors. This is followed by reactivation of the silent X in XX germ cells, but not in the XX somatic cells. The process of reactivation of the silent X chromosome in PGCs is initiated during their migratory journey to the genital ridges and may be cell autonomous. However, substantial X-linked gene reactivation occurs only in response to signals emanating from the somatic compartment of the XX genital ridges and is gender independent (occurring as well in sex reversed embryos).

## Introduction

From early cleavage up to the mid-blastocyst stage, the paternally transmitted X chromosome in mouse embryos is inactive [1,2], with transcripts from the paternal *Xist* gene silencing X-coded genes. By the late blastocyst stage, at embryonic day (E)4,5, the inactive X chromosome (Xi) has reactivated in the inner cell mass [1]. Twenty-four hours later, one of the two X chromosomes has been inactivated by *Xist* transcripts in most of the epiblast cells, randomly with respect to the maternal and paternal chromosomes. By E6.5 virtually all epiblast cells are reported to have undergone X-chromosome inactivation [3,4], leading to monoallelic expression of most X-linked genes.

Mouse primordial germ cells (PGCs) are derived from a small subset of epiblast cells. After specification, the maternal or paternal X chromosome in XX PGCs was shown to be randomly inactivated [5]. Based on a robust statistical analysis of the distribution of two X-linked isoalleles in germline and somatic cells among different embryos, [6] concluded that random X-chromosome inactivation in the PGC precursors and in other epiblast cells had occurred at the same time, as part of the same process [5,6]. A later study, using a LacZ reporter gene integrated into the X chromosome, threw some doubt as to whether PGCs had undergone X-chromosome inactivation prior to germ-cell specification [7]. Recently, early (E6.25) PGC progenitors expressing *Blimp1* have been identified [8]. Using the strong H3K27me3 accumulation indicative of X-chromosome inactivation [9,10], and an X-located green fluorescent protein (GFP)-carrying transgene [11], we show that from their earliest appearance, XX PGC precursors expressing Blimp1 have inactivated one X chromosome, and that X-chromosome inactivation in PGCs is random with respect to parental origin. We followed the process of reactivation of the Xi as the XX PGCs migrate and enter the genital ridges. We conclude that PGCs start the process of X-chromosome reactivation during migration. However signals from the XX urogenital ridge tissue seem to stimulate the resumption of expression of the X-borne GFP transgene in the majority of PGCs between E11.5 and E13.5.

## Results

### X-chromosome Inactivation in XX PGCs and Their Precursors

**Figure 1:**
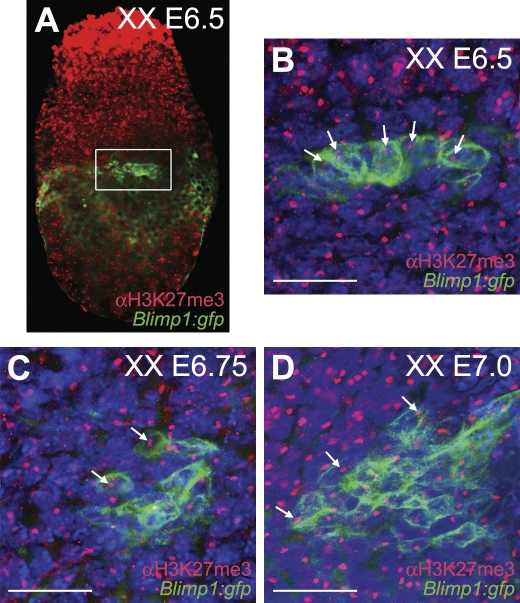
PGC Precursors Show a Pronounced H3K27me3 Nuclear Accumulation Indicative of One Inactive X Chromosome XX *Blimp1:gfp* mouse embryos were immunostained for H3K27me3. GFP under the control of the *Blimp1* promoter was tagged to the cell membrane. (A) E6.5 whole embryo showing in the white box the Blimp1(GFP)-positive PGC progenitors. (B) Magnification of the Blimp1(GFP)-positive PGC progenitor cluster depicted in (A). White arrows indicate individual H3K27me3-positive nuclear accumulation in the PGC progenitors. Note that all somatic cells exhibit a H3K27me3 nuclear accumulation. (C) E6.75 and (D) E7.0 whole embryos similarly stained.

Using transgenic embryos carrying a membrane-tagged GFP driven by the promoter of *Blimp1* [8], we immunostained E6.5, E6.75 and E7.0 embryos with antibody against H3K27me3. At those stages, a nuclear accumulation of H3K27me3, corresponding to the Xi in Blimp1(GFP)-expressing cells was observed (Figure 1), confirming that XX PGC precursors, like their somatic neighbours, were subjected to X-chromosome inactivation from the earliest expression of Blimp1.

**Figure 2:**
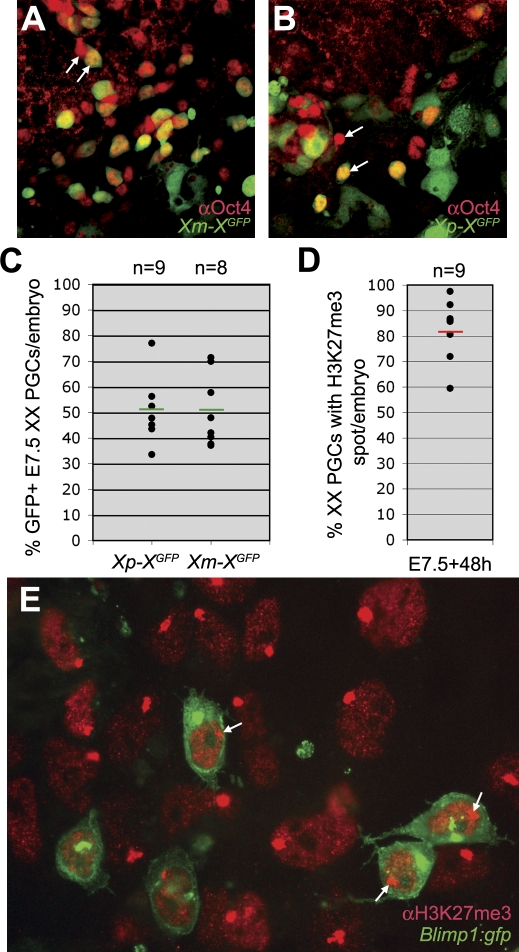
X-chromosome Inactivation in E7.5 PGCs Is Random (A–C) The posterior part of XX E7.5 *X^GFP^* embryos, which inherited X-linked GFP either from the mother (A) or the father (B), were cultured for 48 hours and immunostained for Oct4 to identify the PGCs. White arrows depict PGCs that do or do not co-express GFP. The expression of GFP was analysed in the total number of PGCs per embryo (C). Green bars depict the median, *n* is the total number of embryos analysed. (D,E) The posterior part of XX E7.5 *Blimp1:gfp* embryos were cultured for 48 hours and immunostained for H3K27me3. The total number of Blimp1(GFP)-positive PGCs was screened for the presence of a H3K27me3-positive nuclear accumulation (D) Red bar depicts the median, *n* is the total number of embryos analysed. A representative image of the posterior cluster containing the Blimp1(GFP)-positive PGCs after 48 hours culture is shown in (E). White arrows show Blimp1(GFP)-positive PGCs that contain a prominent H3K27me3 nuclear accumulation.

Shortly after specification (E7.5), the tissue containing the XX PGCs was isolated from embryos carrying a GFP transgene on the X chromosome [11], transmitted either from the mother or from the father, and cultured for 48 hours. 50% of the PGCs expressed GFP, whether the transgene had been transmitted maternally or paternally (Figure 2A–2C), confirming that X-chromosome inactivation was random. Moreover, about 80% of the cultured PGCs contained a prominent H3K27me3 nuclear accumulation (Figure 2D and 2E). Note that the variation in the percentage of GFP-expressing PGCs per embryo was high, reflecting the random nature of the inactivation process.

### X-chromosome Reactivation In Vivo in XX PGCs

**Figure 3:**
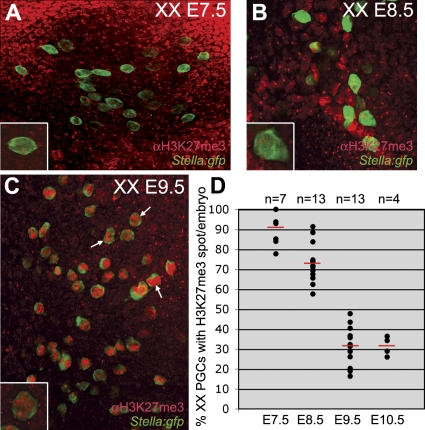
During PGC Migration the H3K27me3 Nuclear Accumulation Is Gradually Lost The total number of PGCs per embryo was analysed in XX *Stella:gfp* embryos after whole mount immunostaining for H3K27me3. Stella is a marker of specified PGCs. (A–C**)** E7.5 (A), E8.5 (B), and E9.5 (C) confocal projections showing all PGCs present in the depicted area. Magnification of single Stella(GFP)-positive PGCs containing a prominent nuclear H3K27me3 accumulation are depicted in the lower right corner of (A–C). Note that the overall levels of H3K27me3 in the nucleus increase during development (white arrows in C). (D) The percentage of PGCs containing a prominent H3K27me3 accumulation from E7.5 to E10.5. Red bars depict the median, *n* is the total number of embryos analysed.

**Figure 4:**
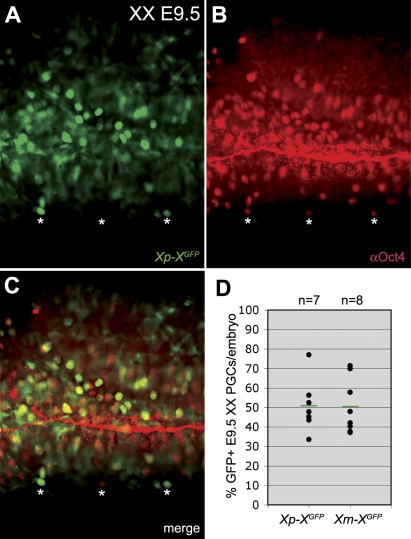
X-chromosome Inactivation in E9.5 PGCs Is Still Random The hindgut of XX E9.5 *X^GFP^* embryos, which inherited X-linked GFP either from the mother or the father, were immunostained whole mount for Oct4 to identify the PGCs. (A–C) White asterisks depict PGCs that do or do not co-express GFP. Note that the lumen of the hindgut shows aspecific Oct4 immunostaining. (D) The expression of GFP was analysed in the total number of PGCs per embryo. Green bars depict the median, *n* is the total number of embryos analysed. Red bar depicts the median, *n* is the total number of embryos analysed.

**Figure 5:**
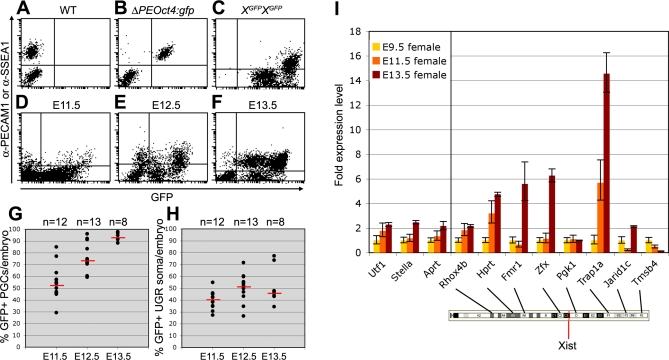
Xi Reactivates in XX PGCs between E11.5 and E13.5 (A,B) FACS-analysis of XX E13.5 WT (A) and Δ*PEOct4:gfp* (B) PGCs show that anti-PECAM1 is a suitable antibody to separate PGCs from the surrounding somatic tissue. Anti-SSEA1 was used to separate XX E11.5 and E12.5 PGCs from the surrounding somatic tissue. (C) FACS-analysis of XX E13.5 XGFP homozygous genital ridges containing 100% GFP-positive cells, used as positive control. (D–F) Representative dot-plots showing FACS-analysis of XX E11.5 (D), E12.5 (E), and E13.5 (F) genital ridges from individual *Xp-X^GFP^* embryos. (G,H) The percentage of FACS-analysed GFP-expressing PGCs (G) and surrounding somatic tissue (H) in the genital ridges of individual XX E11.5, E12.5, and E13.5 *Xp-X^GFP^* embryos. PGCs and somatic cells were respectively positive and negative for SSEA1 (E11.5, 12.5) or PECAM1 (E13.5). Red bars depict the median, *n* is the total number of embryos analysed. (I) Transcriptional levels of X-coded genes and autosomal genes at E9.5, E11.5, and E13.5. Shown are the relative transcription levels of each gene compared to the expression of that same gene observed at E9.5. The localization of the X-coded genes analysed and *Xist* are shown on the cartoon of the X chromosome (from Ensembl).

As the XX PGCs migrated into the endoderm of the invaginating hindgut and subsequently to the genital ridges, the proportion showing a clear H3K27me3 accumulation on the X chromosome decreased although the overall levels of nuclear H3K27me3 were increasing (Figure 3). At E9.5, the percentage of PGCs showing a nuclear H3K27me3 accumulation decreased to about 30%. This suggested that either H3K27me3 is not involved in the maintenance of X-chromosome inactivation or alternatively that the Xi is being reactivated specifically in the PGCs during their migratory journey to the genital ridges. To distinguish between these two possibilities, we analysed the number of E9.5 PGCs expressing the X-linked GFP transmitted either by the father or the mother and observed that the PGCs still showed random X-chromosome inactivation (Figure 4). Once in the genital ridges, the number of PGCs expressing the X-borne GFP transgene increased from just over 50% at E11.5 (random X-chromosome inactivation) to nearly 95% at E13.5, while the somatic cell population over the same period remained at about 50% expression (Figure 5A–5H).

In addition, quantitative reverse transcriptase-polychain reaction (Q RT-PCR) was used to follow the reactivation dynamics of a number of X-chromosomal loci in XX PGCs between E9.5 and E13.5 (Figure 5I). The GFP transgene was integrated relatively close to *Hprt* [12]. Our analysis suggest a pattern of Xi reactivation starting from the centromere to the X-inactivation centre, where *Xist* is localized: *Rhox4b* seems to be already transcribed at E9.5, *Hprt* is reactivated at E11.5, and *Fmr1* and *Zfx* are reactivated only at E13.5 (Figure 5I). These results are in agreement with recent data by [13] using single cell allele-specific RT-PCR. A similar reactivation “wave” from the Xi telomere to the X-inactivation centre should not be excluded. *Jarid1c*, a gene that escapes inactivation [14] was included in our analysis and indeed behaved similarly to the autosomal genes, *Utf1*, *Stella* and *Aprt*. *Trap1a* and *Tmsb4* seem to be developmentally upregulated and downregulated, respectively, between E9.5 and E13.5 in agreement with [15].

Our data together with that of [13] show that during migration both the *Xist*-coating and the H3K27me3 repressive mark on the Xi gradually disappear from the PGCs and the transcription of a few X-coded genes, located near the Xi centromere, is already detectable. This suggests that an early portion of the Xi reactivation process may be intrinsically programmed in the XX PGCs. However, both robust transcription and detectable translational activity, at least from the GFP transgene locus, commences in PGCs only after entering the genital ridges. This suggested the possibility that signals generated in the urogenital ridge (UGR) could be also involved in the reactivation of the X chromosome.

### Is X-Chromosome Reactivation in XX PGCs Induced?

**Figure 6:**
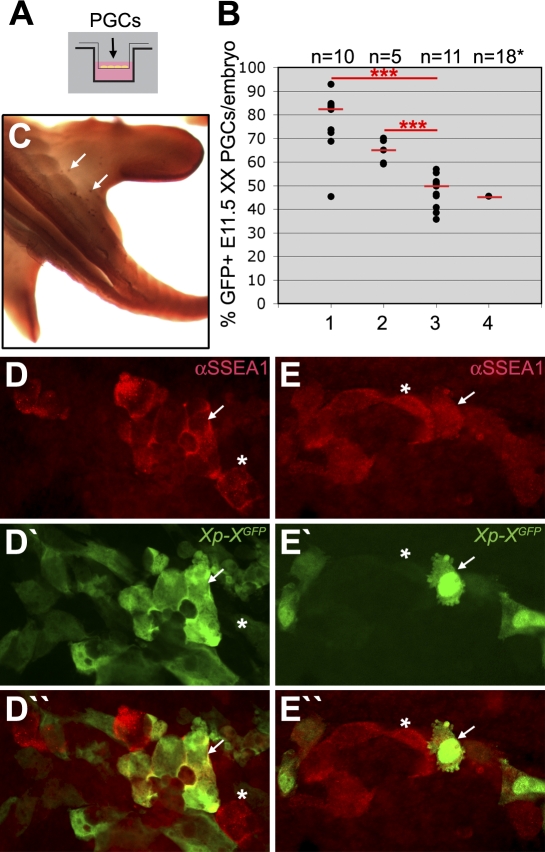
XX E11.5 PGCs Require Somatic XX UGR To Reactivate the Xi (A) A transwell system was used to study the X-chromosome reactivation in XX E11.5 *X^GFP^* PGCs cultured in the top compartment for 48 hours. (B) The percentage of X-linked GFP-expressing PGCs in XX embryos. Genital ridges were dissociated and added to the top compartment (lane 1) or FACS-sorted for SSEA-1, with PGCs (SSEA1-positive) added to the top compartment and somatic tissue (SSEA1-negative) to the lower compartment (lane 2). Lane 3, FACS-sorted PGCs (SSEA1-positive) were added in the top compartment. Lane 4, the base of the tail, containing ectopic PGCs, was dissociated and cultured in the top compartment. Due to the low number of PGCs present ectopically, the results of 18 individual tails analysed were pooled. Red bars depict the median, *n* is the total number of embryos analysed. ***, *p* < 0.001. (C) E11.5 embryo showing ectopic PGCs, positive for alkaline phosphatase-activity (white arrows), in the base of the tail. (D,E) GFP-positive (white arrow) and GFP-negative (asterisk) *Xp-X^GFP^* PGCs (SSEA1-positive) cultured together with SSEA1-negative somatic tissue (D) or with culture medium alone (E).

To experimentally clarify whether signals from the UGR environment were required to achieve substantial Xi reactivation, we used a transwell system (Figure 6A), in which X-borne GFP transgenic PGCs were cultured in the top compartment with or without UGR somatic cells. The proportion of PGCs showing expression of the X-borne GFP transgene in the course of a 48-hour culture period was then studied (Figure 6B–6E). E11.5 fluorescence-activated cell sorting (FACS)-sorted PGCs failed to undergo Xi reactivation in culture medium alone (Figure 6B, lane 3 and Figure 6E), but when E11.5 UGR tissue from XX embryos was present in the transwell in the top (Figure 6B, lane 1 and Figure 6D) or lower (Figure 6B, lane 2) compartment, the percentage of PGCs that expressed the GFP transgene increased significantly. In addition, E11.5 ectopic XX PGCs present in the base of the tail (Figure 6C) cultured with their surrounding somatic tissue failed to reactivate after 48 hours (Figure 6B, lane 4).

**Figure 7:**
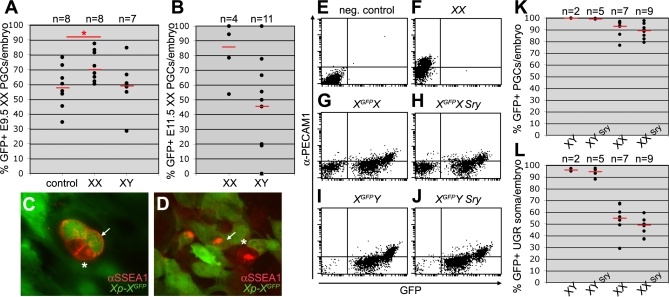
XX, but Not XY, UGRs Induce Xi Reactivation in E9.5 and E11.5 XX PGCs A transwell system was used to study the X-chromosome reactivation in E9.5 and E11.5 *X^GFP^* PGCs cultured for a period of 48 hours. (A) The percentage of X-linked GFP-expressing XX E9.5 PGCs after culture. Hindgut and the surrounding mesentery were dissociated and cultured in the top compartment with the lower compartment containing: medium only (control), one dissociated XX E11.5 UGR pair (XX), or one dissociated XY E11.5 UGR pair (XY). Red bars depict the median, *n* is the total number of transwell filter membranes (each containing 1.5× embryo) analysed. *, *p* < 0.05. (B) The percentage of X-linked GFP-expressing XX E11.5 PGCs after culture. FACS-sorted PGCs (SSEA1-positive) were cultured in the top compartment with FACS-sorted XX (XX) or XY (XY) E11.5 UGR somatic tissue (SSEA1-negative). Red bars depict the median, *n* is the total number of embryos analysed. (C,D) GFP-positive (white arrow) and GFP-negative (asterisk) *Xp-X^GFP^* E9.5 PGCs (SSEA1-positive) after culture. (E–J) Representative dot-plots showing FACS-analysis of E13.5 genital ridges from individual *XX* (E,F), *X^GFP^X* (G), *X^GFP^X Sry* (H), *X^GFP^Y* (I), and *X^GFP^Y Sry* (J) embryos. (K,L) The percentage of FACS-analysed GFP-expressing PGCs (K) and surrounding somatic tissue (L) in the genital ridges of individual *X^GFP^X*, *X^GFP^X Sry*, *X^GFP^Y*, and *X^GFP^Y Sry* E13.5 embryos. PGCs and somatic cells were respectively positive and negative for PECAM1. Red bars depict the median, *n* is the total number of embryos analysed.

Are the PGCs programmed to respond to the UGR somatic signals only at a particular point in their development? To answer this question migrating XX PGCs from E9.5 hindgut were placed in the transwell top compartment for 48 hours. When exposed only to E9.5 hindgut tissue, no reactivation occurred; but when E11.5 XX UGR tissue was added (in the lower compartment), again around 70% of the PGCs expressed the transgene (Figure 7A–7D) after a 48-hour culture period. By contrast, addition of E11.5 XY UGR tissue had no effect. Thus, while E11.5 XX PGCs in vivo had barely started to reactivate (expressing X-coded GFP protein), at the equivalent stage (E9.5 + 48 hours culture) the hindgut PGCs had undergone reactivation to a level similar to E11.5 FACS-sorted XX PGCs cultured under similar conditions*.* These results indicate not only that signal(s) from the XX UGR are sufficient to stimulate the progression of Xi reactivation, but also that XX PGCs at E9.5 have already acquired competence to respond to those signal(s). The latter is consistent with our results that the H3K27me3 nuclear enrichment is absent in most E9.5 XX PGCs.

To confirm that the XY E11.5 UGR indeed had no effect reactivating the X-linked GFP transgene, we cultured in the top transwell compartment FACS-sorted E11.5 XX PGCs together with either FACS-sorted XX or XY UGR somatic cells for 48 hours. In general, we obtained per UGR pair 1.000 PGCs and 100.000 somatic cells independently of the sex. PGCs showed reactivation when cultured with XX, but not with XY E11.5 UGR somatic tissue (Figure 7B). Next, we studied whether it is the gender or the sex chromosome constitution of the gonad that is responsible for Xi reactivation. To do this we re-evaluated the X-chromosome activity in the germ cells of sex-reversed XX E13.5 embryos. At E14.5, the ratio of X-coded HPRT to the autosomal-coded APRT increased in XX PGCs independently of the gender [16]. Here, XY males carrying a *Sry*-coding autosomal transgene combined with the deletion of the *Sry* gene from the Y chromosome [17] were crossed with XX^GFP^ females. From the GFP-positive embryos obtained, 25% should be XX and contain the autosomal-coded *Sry* and therefore be phenotypically males and 25% should be XX (phenotypically females). The XY GFP-positive embryos should be phenotypically males (if containing the autosomal-coded *Sry*) or females. E13.5 embryos were isolated and their gonads individually analysed by FACS (Figure 7E–7L). The data clearly showed that Xi reactivation occurred in E13.5 XX PGCs independently of the presence of the *Sry*-transgene and therefore independently of the gender. We conclude that signals from XX UGR somatic tissue (independently of the gender) are important for the reactivation of Xi.

### X-Chromosome Reactivation during Derivation of EGCs

**Figure 8:**
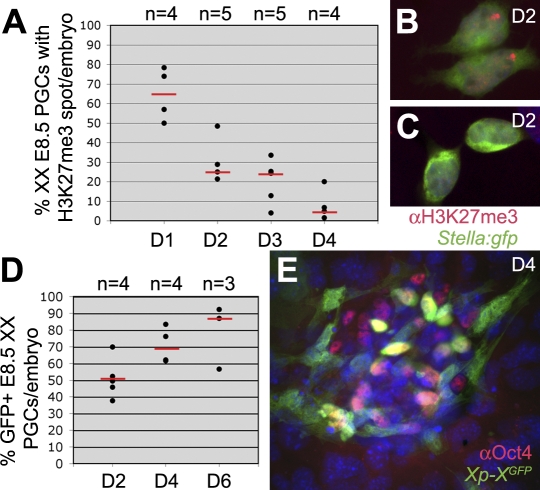
Kinetics of Xi Reactivation during EGC Derivation from XX E8.5 PGCs (A) The percentage of XX *Stella:gfp* PGCs containing a prominent H3K27me3 nuclear accumulation during the first 4 days (D1-D4) of EGC derivation. Red bars depict the median, *n* is the total number of embryos analysed. (B,C) Stella(GFP)-positive PGCs after 48 hours culture towards EGCs, showing a prominent H3K27me3 nuclear accumulation and a migratory phenotype (B) or rounding up and containing no H3K27me3 accumulation or nuclear staining (C). (D) The percentage of GFP-expressing XX E8.5 *Xp-X^GFP^* PGCs (Oct4-positive) per embryo during the first 6 days (D) of EGC derivation. Red bars depict the median, *n* is the total number of embryos analysed. (E) Oct4-positive PGCs after 4 days of culture showed a mixed population of GFP-positive and GFP-negative cells.

When E8.5 XX PGCs were cultured on Sl^4^-m220 feeders in EGC-medium [18] to escape meiosis entry, but instead to give rise to pluripotent self-renewing EGCs, we observed that the proportion of cells showing an H3K27me3 nuclear accumulation declined rapidly (Figure 8A–8C). In contrast to PGC development in vivo, we did not observe an upregulation of uniform nuclear H3K27me3 staining during the derivation of EGCs from XX E8.5 PGCs. However, as in vivo, the decline of the H3K27me3 nuclear accumulation was followed by an increase in the expression of the X-borne GFP transgene, indicative of Xi reactivation (Figure 8D and 8E). We conclude that at early stage of EGC-derivation from E8.5 XX PGCs, the Xi is also reactivated over a period of several days, presumably in response to signals from the feeders or factors present in the culture medium.

## Discussion

### X-Chromosome Inactivation Occurs in All Epiblast Cells during Implantation

Almost 30 years ago Rastan and colleagues determined that X-chromosome inactivation took place in all epiblast cells in metaphase [3,4]. However, to date it has been unclear whether the PGC precursors divide at all [19] and therefore whether these PGC precursors would indeed undergo X-chromosome inactivation. We used an antibody to H3K27m3 to show that inactivation of one X chromosome in XX embryos had occurred by E6.5, in all epiblast cells expressing *Blimp1* and hence destined to become PGCs, as well as in their *Blimp1*-negative epiblast neighbours. Moreover, the inactivation of an X-borne transgene in the specified PGCs was clearly random with respect to parental origin. Our results thus show that PGCs, like other epiblast-derived cells, are subjected to random X-chromosome inactivation.

### Relationship between H3K27me3, X-Chromosome Reactivation, and Global Repression of Transcription

In XX embryos, as PGC migration proceeds, the level of H3K27m3 in the nucleus as a whole was seen to increase, confirming previous findings [20]. In contrast, the proportion of PGCs showing a prominent H3K27me3 accumulation on the Xi declined drastically between E7.5 and E9.5. The H3K27me3 nuclear accumulation could still be detected even if against the global staining background of H3K27me3. In agreement, the percentage of E9.5-E10.0 PGCs showing no *Xist* accumulation on the Xi drop to 50–70% [13,21], whereas reactivation of Xi-coded genes distant from the *Xist* locus was observed. Our data combined with [13,21] favors the hypothesis that Xi reactivation in PGCs is initiated as part of their epigenetic reprogramming during migration to the genital ridges. At the same time, a general wave of chromatin repression (overall high levels of H3K27me3) ensures global transcriptional repression as the PGCs enter the genital ridges.

In any case, reactivation of the X-borne GFP transgene in female PGCs occurred between E11.5 and E13.5, resembling in timing the reactivation of *Hprt* detected biochemically by [22]. In agreement, the GFP transgene is integrated in the proximal region of the X chromosome, relatively close to *Hprt* [12].

### Reactivation of Xi in the URG Is Stimulated by Factor(s) Secreted by the XX URG Soma

Our transwell studies indicated that reactivation of the GFP transgene was stimulated by signals emanating from the somatic tissue of the XX UGR at E11.5. Some reactivation was induced when the XX somatic tissue was separated from the PGCs by the transwell filter, but the level of reactivation was higher when XX somatic cells and PGCs were cultured together. This indicated either that the signalling molecule is diffusible and dose-dependent, or that cell-cell contact increases the inducing effect.

Moreover, XX sex-reversed PGCs developing in a phenotypically male XX genital ridge showed clear Xi reactivation (our data and [16]), suggesting that XX URGs, independently of the gender, are important for the reactivation of the Xi. Two recent screens have determined the expression profile of the somatic compartment of the UGRs from E10.5 to E13.5 [23,24]. It would be interesting to perform a similar screen using XX sex reversed UGR soma and to compare it to the XX and XY UGR soma-expression profiles to understand the signals that may be involved in the later part of the Xi reactivation process.

Finally, an increase in the levels of X-linked GFP during derivation of E8.5 EGCs was observed after 4 days of culture, instead of the 2 days needed to induce X-linked GFP to similar levels in E9.5 or E11.5 PGCs by E11.5 XX UGRs. It is therefore unclear whether the same mechanism that regulates Xi reactivation in vivo is involved in the reactivation of Xi during the derivation of EGCs.

### Meiotic Entry versus X-chromosome Reactivation

E11.5 PGCs exposed to the XX UGR environment (independent of the gender) reactivated the Xi, whereas ectopic PGCs in culture or in vivo [7] did not. By contrast, entry of XX germ cells into meiosis occurs in vivo within days of entry into the genital ridge, and with the same timing in ectopic sites, in organ-cultured lung aggregates and in tissue culture (reviewed in [25]).

Moreover, during the derivation of EGCs, which escape meiotic entry but keep on self-renewing instead, X-chromosome reactivation clearly occurs (our data and [26]); and the XX sex-reversed PGCs undergo Xi reactivation uniformly and follow the male-pathway (mitotic arrest) [27,28]. Together our findings support the idea that the reactivation of Xi and meiosis may not be linked although the relation between X-chromosome reactivation and meiotic entry remains unclear.

## Materials and Methods

### Embryos and PGC isolation.

Wild type (F1), Δ*PEOct4:gfp* [29], *Blimp1:gfp* [8] and *Stella:gfp* [30] were on a mixed background C57BL/6 and CBA; *X^GFP^* is the D4/XEGFP transgenic line [11]; *XYTdym1Sry* is from [17] and genotyping was done according to [31]. Noon of the day of the vaginal plug was designated E0.5. Embryos were collected in cold DMEM (Invitrogen) supplemented with 7.5% fetal calf serum (FCS) and 10 mM HEPES. We isolated the posterior region containing the PGCs of E7.5 and E8.5 embryos; the entire hindgut containing the surrounding mesenterium of E9.5 embryos; the region containing both UGRs (and including the dorsal aorta) of E10.5 and E11.5 embryos; the genital ridges of E12.5 and E13.5 embryos.

### PGC culture.

E7.5 PGC clusters were cultured for 48 hours on DMEM+15% FCS on fibronectin (20 μg/ml) coated glass coverslips in 4-well culture plates (NUNC) at 37 °C and 5% CO_2_ on air. E9.5 hindguts, E11.5 UGRs and E11.5 tails were trypsinysed 15 minutes at 37 °C, ressuspended and an equal amount of FSC added to the cell suspension. After spinning down (3,000 rpm for 3 minutes), the cells were ressuspended in Knockout^TM^ DMEM (Invitrogen) containing 20% Knockout^TM^ serum replacement (Invitrogen) and 0.1 μg/ml bFGF (Invitrogen). Cells were seeded at a density of 1.5 × E9.5 hindgut/24-well, 1 × E11.5 UGR pair/24-well and 1 × E11.5 tail/12-well in the upper or lower compartment of the transwell (0.4 μm pore size, polycarbonate membrane, Costar) and cultured 48 hours at 37 °C and 5% CO_2_ on air.

For E8.5 XX EGC derivation, the posterior region of E8.5 embryos was trypsinysed as above and seeded on coverslips coated with Sl^4^-m220 feeders and cultured as described [18] on in 4-well culture plates (NUNC).

### FACS sorting and FACS analysis.

E11.5 PGCs and somatic tissue were separated by FACS-sorting for culture. In this case, *X^GFP^* female UGRs were pooled and after trypsinysation (see above) the cells were incubated with mouse anti-SSEA1 antibody 15 minutes on ice, washed 3× in PBS containing 0.1% bovine serum albumin (BSA, Invitrogen), incubated with goat anti-mouse IgM (Molecular Probes) 15 minutes on ice, washed 3× in PBS containing 0.1% BSA and used for FACS-sorting (FACSAria, BD Bioscience). PGCs and somatic cells were ressuspended and seeded as above. Using FACS-sorting, we obtain in average 1000 PGCs and 100000 somatic cells per E11.5 UGR pair.

Individual E11.5, E12.5 and E13.5 genital ridge-pairs from wild type (F1), Δ*PEOct4:gfp, X^GFP^* and *X^GFP^ Sry* (from *XYTdym1Sry* male x XX^GFP^ female crosses) embryos were isolated, immunostained with mouse anti-SSEA1 or R-Phycoerythrin-conjugated rat anti-PECAM1 (1:250, BD Biosciences) as above, but using PBS containing 0.1% BSA and 0.1% NaAzide, and the percentage of GFP-positive/negative cells was analysed by FACS (FACSAria, BD Bioscience).

### Q RT-PCR.

E9.5, E11.5 and E13.5 *Xp-X^GFP^* female embryos were isolated, immunostained with mouse anti-SSEA1/rat anti-Pecam1 and FACS-sorted as above. Total RNA isolated from 1000 PGCs using RNeasy Micro Kit (Qiagen) was amplified according to the single-cell method [32] with these modifications: instead of seeding single cells, total RNA was diluted to the equivalent of 5–10 cells per sample (75–150 pg), and four to six replicates of each sample were amplified; spike RNAs were not used, but replaced with nuclease-free water; the T7 promoter addition step was not performed. The cDNA (diluted 1/40 in nuclease-free water) were analysed by Q RT-PCR after the 20-cycle amplification step. Q RT-PCR was performed on an ABI Prism 7000 Sequence Detection System using SYBR Green chemistry. The PCR reaction (20 μl) containing 1x SYBR Green (Sigma) master mix and 1 μM of each primer were run in duplicate. Gene expression was normalised to *Gapdh* levels, and quantification calculated using the ΔΔCt method. Primers were designed with the Primer3 algorithm [33]. Primer sequences were as follows: *Rhoxb4* (NM_021300, TGGAAGGGGACAAAGCAGAA and CCTGGTCCTCTTGGTTGCTG), *Hprt* (NM_013556, CCTGTGGCCATCTGCCTAGT and GGGCGCAGCAACTGACATT), *Fmr1* (NM_008031, TGAATTCAGCAAGGCGCTAAC and CTCCAACTCCATTTGAAAAGATGTG), *Zfx* (NM_001044386, CGTCCTTCAGTGCACGTCTG and ATGCAACACTGGGGGCTATG), *Pgk1* (NM_008828, CACTTGCTGTGCCAAATGGA and CCAGTGCTCACATGGCTGAC), *Trap1a* (NM_011635, AGCTGAGGAAATGGGTGCTG and ATGATGGCCAGGAACACAGG), *Jarid1c* (NM_013668, AGGCTTTTGGTGCTTGTCCA and ACAGCATGAACCCAGGAGGA), *Tmsb4* (NM_021278, GCTGGCGAATCGTAATGAGG and AGGCAATGCTCGTGGAATGT), *Utf1* (NM_009482, TTGCTCCCCAGTCGTTGAAT and CAGAGGGCCCAGAACTGTTG), *Stella* (NM_139218, AACGGGACAGTGAGCCATTC and CCCGATTTTCGCATTCTCAG), *Aprt* (NM_009698, TATGGGAAGGCTGAGCTGGA and GCCAGGAGGTCATCCACAAT) and *Gapdh* (AK144690, CATGGCCTTCCGTGTTCCT and GCGGCACGTCAGATCCA).

### Immunofluorescence, statistics, and PGC counting.

Immunostaining on whole mount embryos, coverslips and transwell filter membranes was preformed as described [30]. The antibodies used were using rat anti-GFP (1:500, Nacalai Tesque), rabbit anti-H3K27me3 (1:1,000), mouse anti-Oct4 (1:200, BD Biosciences) and secondary antibodies were from Molecular Probes. After immunostaining, quantitative analysis was performed on the total number of PGCs per embryo/coverslip/transwell using an Olympus IX71 (London, UK) inverted microscope or a Bio-Rad Radiance 2000 confocal microscope (CA, USA). Alkaline phosphatase-activity staining was preformed as described [34]. Statistical analysis was done using the Student's t-test.

## Abbreviations

E: embryonic day
FACS: fluorescence-activated cell sorting
GFP: green fluorescent protein
H3K27me3: trimethylation of histone 3 on lysine 27
PGCs: primordial germ cells
UGRs: urogenital ridges
Xi: inactive X chromosome
